# A Potential Role of Myeloid DAP12-Associating Lectin (MDL)-1 in the Regulation of Inflammation in Rheumatoid Arthritis Patients

**DOI:** 10.1371/journal.pone.0086105

**Published:** 2014-01-21

**Authors:** Der-Yuan Chen, Ling Yao, Yi-Ming Chen, Chi-Chen Lin, Kui-Chou Huang, Szu-Ting Chen, Joung-Liang Lan, Shie-Liang Edmond Hsieh

**Affiliations:** 1 Faculty of Medicine, National Yang-Ming University, Taipei, Taiwan, R.O.C.; 2 Division of Allergy, Immunology and Rheumatology, Taichung Veterans General Hospital, Taichung, Taiwan, R.O.C.; 3 Institute of Biomedical Science, National Chung-Hsing University, Taichung, Taiwan, R.O.C.; 4 Institute of Microbiology and Immunology, Chung Shan Medical University, Taichung, Taiwan, R.O.C.; 5 Infection and Immunity Research Center, National Yang-Ming University, Taipei, Taiwan, R.O.C.; 6 Department of Orthopedics and Traumatology, Taichung Veterans General Hospital, Taichung, Taiwan, R.O.C.; 7 Division of Immunology and Rheumatology, China Medical University Hospital, Taichung, Taiwan, R.O.C.; 8 College of Chinese Medicine, China Medical University, Taichung, Taiwan, R.O.C.; 9 Institute of Clinical Medicine, National Yang-Ming University, Taipei City, Taiwan; University Hospital Jena, Germany

## Abstract

The pathogenic roles of myeloid DAP12-associating lectin-1(MDL-1) and DAP12 in human rheumatoid arthritis (RA) remain unknown. Frequencies of MDL-1-expressing monocytes in 22 active RA patients, 16 inactive RA patients, 12 osteoarthritis (OA) patients and 10 healthy controls (HC) were determined by flow-cytometry analysis. The mRNA expression levels of MDL-1 and DAP12 on PBMCs were evaluated by quantitative PCR, and their protein expression levels in the synovium were examined by immunohistochemistry. Significantly higher median percentages of circulating MDL-1-expressing monocytes were observed in active RA patients (53.6%) compared to inactive RA patients (34.1%), OA patients (27.9%), and HC (21.2%). Levels of MDL-1 and DAP12 gene expression in PBMCs and their protein expression in the synovium were significantly higher in active RA patients than in inactive RA or OA patients. MDL-1 levels were positively correlated with parameters of disease activity, articular damage, and levels of proinflammatory cytokines. MDL-1 activator (Dengue virus type 2 antigen) stimulation on PBMCs resulted in significantly enhanced levels of proinflammatory cytokines in RA patients compared to those in OA patients or HC, indicating that MDL-1 activation is functional. Frequencies of MDL-1-expressing monocytes and levels of MDL-1 and DAP12 gene expression significantly decreased after effective therapy. Concordant overexpression of MDL-1 and DAP12 were correlated with increased production of proinflammatory cytokines in RA patients, suggesting their roles in regulating articular inflammation.

## Introduction

Rheumatoid arthritis (RA) is a chronic inflammatory disease characterized by the infiltration of macrophages and T cells into the joints, synovial hyperplasia, and bone erosions associated with excessive osteoclast activity [1]–[2]. Results of recent studies show that the osteoclastogenesis induced by receptor activator of NF-κB (RANK)-RANK ligand (RANKL) is augmented through the immunoreceptor tyrosine-based activation motif (ITAM)-harboring signaling pathway [3]–[4]. In addition, macrophage-derived proinflammatory cytokines such as tumor necrosis factor (TNF)-α, interleukin (IL)-1β, and IL-6, are crucial mediators in rheumatoid synovitis and subsequent bone destruction in RA [5]–[6]. IL-17A can stimulate monocytes to produce proinflammatory cytokines, and thus amplify the inflammatory cascade [7]. Additionally, enhanced expression of IL-17 has been observed in rheumatoid synovium [8]. Accumulating evidence shows that alterations in proinflammatory cytokines are viewed as both a possible important pathogenic factor and a potential target for therapeutic intervention in RA [9]–[13].

The ITAM-dependent signaling pathway is an important “co-stimulatory” pathway for RANKL-dependent regulation of bone remodeling [14]–[15]. DNAX activation protein 12 (DAP12), a type I transmembrane homodimer [16], is a novel ITAM-bearing signaling glycoprotein that has been implicated in immune responses and in osteoclast formation [17]–[18]. DAP12 expression has also been demonstrated to be increased in the synovium of active RA patients [19]. Myeloid DAP12-associated lectin-1 (MDL-1) is a C-type lectin domain family 5-member A (CLEC5A) and contains a charged residue in the transmembrane region that enables it to bind with DAP12 [20]–[21]. MDL-1 associates non-covalently with adaptor DAP12 to form receptor complexes involved in inflammatory responses [21]. Recent studies show that MDL-1 is highly expressed on TNF-activated monocytes [20] and acts as a key regulator of synovitis and bone erosion in murine arthritis [22]. Cross-linking of MDL-1 receptors induces DAP12-ITAM-dependent calcium mobilization [17], [20] and activation of spleen tyrosine kinase (Syk) [4], [17].

Recently, the roles of MDL-1 and its associated adaptor-DAP12 were highlighted in a study of murine arthritis [22]. Joyce-Shaikh *et al*. demonstrated that activation of MDL-1 receptors during joint inflammation enhanced myeloid cell infiltration and promoted expression of proinflammatory cytokines including IL-1β, IL-6, IL-17A, and TNF-α, resulting in cartilage damage and bone erosion [22]. In contrast, the functional blockade of MDL-1 receptor via MDL-1 deletion reduced the clinical signs of murine arthritis [22]. Chen *et al*. also observed that the knockdown of MDL-1/CLEC5A suppressed the release of proinflammmatory cytokines by Dengue virus (DV)-infected macrophages [23]. These results suggest that therapeutic targeting of the MDL-1 receptor may inhibit both synovitis and the bone destructive pathway during inflammatory conditions. However, there is no data concerning the role of MDL-1 in the pathogenesis of human RA.

In this study, we investigated the percentages of circulating MDL-1-expressing monocytes using flow-cytometry analysis, and analyzed serum levels of markers for systemic inflammation and proinflammatory cytokines in RA patients, OA patients, and healthy control subjects. The mRNA expression levels of MDL-1 and its associated adaptor-DAP12 on peripheral blood mononuclear cells (PBMCs) were examined by quantitative PCR (qPCR). In addition, the expression levels of MDL-1 and DAP12 in synovial membrane (SM) were determined by immunohistochemistry (IHC). The relationship between MDL-1 expression levels and parameters of disease activity was also evaluated for all RA patients.

## Materials and Methods

### Subjects

A total of 38 consecutive patients who fulfilled the 2010 RA classification criteria of the American College of Rheumatology (ACR)/European League Against Rheumatism (EULAR) collaborative initiative [24] were enrolled in this study. Early RA was defined as RA with a symptom duration≦2 years [25]. Disease activity was assessed by the 28-joint disease activity score (DAS28) [26]. Inactive RA, known as RA with low disease activity, is defined as a DAS28≦3.2 [27]. Radiographs of both hands and feet were assessed for erosions and joint space narrowing (JSN) using the Genant modified total Sharp score (range 0–290) [28] by two independent readers blinded to laboratory data. Twelve patients who fulfilled the 1986 ACR criteria for knee osteoarthritis (OA) [29] were included as controls. Ten age-matched healthy volunteers, who had no rheumatic disease, were used as normal controls.

### Ethics Statement

This study was approved by the Institutional Review Board of Taichung Veterans General Hospital, and written consent was obtained from all participants according to the Declaration of Helsinki.

### Quantitation of MDL-1-expressing Cells Using Flow Cytometry Analysis

To quantify expression levels of MDL-1, 1000 µl samples of whole blood were obtained and stained with phycoerythrin (PE)-conjugated anti-MDL-1 mAb (R&D Systems, Minneapolis, MN, USA) and Phycoerythrin-Cyanin 5 (PC5)-conjugated anti-CD14 mAb (Beckman Coulter, Brea, CA, USA) according to protocols of the respective manufacturers. Fluorescent antibodies, mouse IgG2b-PE (R&D Systems, Minneapolis, MN, USA) and IgG2a-PC5 (Beckman Coulter, Brea, CA, USA) were used as isotype controls. After incubation for 20 minutes in the dark at room temperature, cells were reacted with 500 µl of OptiLyse C Lysis Solution (Beckman Coulter, USA) for 10 minutes to lyse red blood cells. Then, 500 µl PBS was added into each tube to stop the reaction prior to flow cytometry (Beckman Coulter, Brea, CA, USA) analysis. Monocytes were gated on the basis of CD14+/side scatters (SSC) and at least 2 x10^5^ total cells from each sample were analyzed.

### Quantitative PCR Analysis for mRNA Expression Levels of MDL-1, DAP12, and Proinflammatory Cytokines on PBMCs

PBMCs were immediately isolated from venous blood using Ficoll-Paque™ PLUS (GE Healthcare Biosciences AB, Uppsala, Sweden) density gradient centrifugation. Total cellular RNA was obtained from PBMCs by the guanidinium isothiocyanate method [30]. RNA was quantitated by spectrophotometry at 260 nm. A 2.5 µg RNA aliquot was reverse transcribed with 200U of Moloney murine leukemia virus reverse transcriptase (Fermentas, Thermo Fisher Scientific Inc., MD, USA). The qPCR was performed using the IQ^2^ Fast qPCR System (Bio-genesis Technology Inc., Taipei, Taiwan) with a method modified from that described in previous reports [31]–[32]. The primers utilized in this study were adapted and redesigned based on information published on websites. The following are the oligonucleotide primers used for MDL-1, sense primer 5′-CAATTGTCAACACGCCAGAG-3′ and antisense primer 5′-GTC GCACAGTTGAAATTCTG-3′; DAP12, sense primer 5′-AGCGATTGCAGTTGCTC TAC-3′ and antisense primer 5′-GTGATACGCTGTTTCCGGGT-3′; IL-1β, sense primer 5′-GCTGATGGCCCTAAACAGATGAA-3′ and antisense primer 5′-TGA AGCCCTTGCTGTAGTGGTG-3′; IL-6, sense primer 5′-AAGCCAGAGCTGTC AGATGAGTA-3′ and antisense primer 5′-TGT CCTGCAGCCACTGGTTC-3′; IL-17A, sense primer 5′-TGTCCACCATGTGGCCTAAGAG-3′ and antisense primer 5′-GTCCGAAATGAGGCTGTCTTTGA-3′; TNF-α, sense primer 5′-CCACTTCGA AACCTGGGATTC -3′ and antisense primer 5′-TTAGTGGTTGCCAGCACT TCA-3′; the housekeeping gene GAPDH, sense primer 5′-GAAGGTGAAGGTC GGAGTC-3′ and antisense primer 5′-GAAGATGGTGATGGGATTTC-3′. PCR was performed in a total volume of 10.0 µL containing 10 ng of cDNA, 5 µL 2x IQ2 Fast qPCR System Master Mix, 0.375 µL of each oligonucleotide primer, and RNase-free water. Amplification cycles were performed at 95°C for 10 min, followed by 40 cycles of denaturation at 95°C for 10 s, annealing and extension at 60 °C for 30 s. The purity of PCR products was assessed by dissociation curve plots. To standardize mRNA expression levels of MDL-1 and DAP12, the expression levels of the housekeeping gene GAPDH were determined in parallel with each sample. The relative expression levels of MDL-1 and DAP12 were calculated using the comparative threshold cycle (Ct) method and evaluated using 2^−ΔΔCt^, where ΔΔCt = Patent (Ct _target gene_–Ct _GAPDH_)–Mean of controls (Ct_ target gene_–Ct _GAPDH_) [33].

### Determination of Levels of Proinflammatory Cytokines Using ELISA

Serum levels of TNF-α, IL-6, and IL-17A were determined in 22 active RA patients, 16 inactive RA patients, 12 OA patients and 10 healthy control subjects using ELISA (PeproTech Inc., Rocky Hill, NJ, USA) according to the manufacturer’s instructions. IL-1β levels were determined using ELISA (RayBiotech Inc., Norcross, GA, USA). Levels of supernatant cytokine were determined using the aforementioned ELISA.

### Ex Vivo Study to Elucidate Factors Affecting the Expression of MDL-1 and DAP12

Joyce-Shaikh [22] reported that TNF-α but not interferon (IFN)-γ promoted MDL-1 expression in murine arthritis. To elucidate probable factors affecting MDL-1 expression in human RA, we examined changes in percentages of circulating MDL-1-expressing monocytes, and changes in mRNA expression levels of MDL-1 and DAP12 on PBMCs treated with TNF-α (5 µg/ml, R&D Systems Inc., Minneapolis, MN, USA) and IL-1β (5 µg/ml, R&D Systems Inc.) using a method modified from previous reports [22].

### Ex Vivo Induction of Cytokines on PBMCs Treated with MDL-1 Activator

To explore the functional role of MDL-1 activation which may have a possible link with DAP12 and inflammatory response in human RA; we examined changes in mRNA expression levels and supernatant levels of downstream cytokines on PBMCs treated with MDL-1 activator using a method modified from previous reports [34]. Based on results of a previous study showing Dengue virus is a potent activator of MDL-1 [23], we used Dengue virus type 2 (DV2)-antigen (20 µg/ml, MyBioSource Inc., San Diego, CA, USA) as the MDL-1 activator [35]. PBMCs were obtained from 6 active RA patients, 5 OA patients, and 5 healthy volunteers, and were re-suspended in RPMI 1640 medium (Thermo Fisher Scientific Inc., Waltham, MA, USA) supplemented with 100 units/ml penicillin, 100 µg/ml streptomycin, and 10% fetal blood serum at a final concentration of 1×10^6^ cells/well. PBMC samples were incubated at 37°C in a 5% CO_2_ humidified atmosphere for 24 h in the absence or presence of the MDL-1 activator, DV2-antigen (20 µg/ml, MyBioSource Inc.). Changes in mRNA expression levels of MDL-1, DAP12, and downstream cytokines, including IL-1β, IL-6, IL-17A, and TNF-α were determined by the aforementioned method. The cell-free supernatant was harvested, and levels of IL-1β, IL-6, IL-17A, and TNF-α were determined by the aforementioned method.

### Immunohistochemistry for MDL-1 and DAP12 Expression on SM

Synovial tissue specimens were obtained during joint replacement surgery or arthroscopic synovectomy for knee arthritis. Immunostaining for MDL-1 and DAP12 on SM was performed as previously described [19] with modifications on samples obtained from 15 active RA patients, 14 inactive RA patients, and 12 OA patients. Paraffin-embedded samples were cut into 4-µm sections, incubated to assist binding to slides, and then deparaffinized in xylene for a total of 20 minutes at 56°C, followed by rehydration through a series of descending ethanol concentrations. Peroxidase blocking with 3% hydrogen peroxide for 10 min and protein blocking with 2% horse serum for 20 min at room temperature were performed. The sections were then washed with PBS and overlaid overnight with a goat monoclonal antibodies against MDL-1 (Abcam, Cambridge, UK) and DAP12 (Santa Cruz Biotechnology, CA, USA) at concentrations of 2 µg/ml. To identify the cell types that express MDL-1 and DAP12 in synovial membrane, serial sections were stained with mouse monoclonal antibodies against CD68 (Leica Microsystems, Wetziar, Germany) and mouse monoclonal Ab against CD15 (Leica Microsystems). After washing with PBS, binding of the secondary polymer/HRP anti-goat IgG (Nichirei Biosciences, Tokyo, Japan) was detected using an immunodetection kit (Lab Vision Corporation, Fremont, CA, USA). Negative controls were obtained by using the mouse IgG1 isotype control antibody. Appropriate positive controls were used throughout the study. Then, the specimens were counterstained with Mayer’s hematoxylin, and images were captured with a LEICA DMRBE microscopic/Pixera Digital Camera System (Leitz Microsystems, Wetzlar, Germany). The data of positive staining for MDL-1 and DAP12 within the SM were expressed as mean value/SM cross section area.

### Determination of Serum Levels of C-reactive Protein (CRP), Rheumatoid Factor (RF)-IgM and Anti-cyclic Citrullinated Peptide (Anti-CCP) Antibody

Serum levels of CRP and RF-IgM were measured by nephelometry (Dade Behring Inc. Newark, DE, USA). Determination of anti-CCP antibody was performed by ELISA using a commercial kit (INOVA Diagnostics Inc., San Diego, CA, USA).

### Statistical Analysis

Results are presented as the mean ± standard deviation (SD) or median (interquartile range). The nonparametric Kruskal-Wallis test was used for comparisons between groups. When this test showed a significant difference, the exact P-value was determined using the Mann-Whitney U test. The correlation coefficient was calculated using the nonparametric Spearman’s rank correlation test. The Wilcoxon signed rank test was employed to compare levels of circulating MDL-1-expressing monocytes and the mRNA expression levels of MDL-1 as well as DAP12 during follow-up in RA patients after therapy. A P-value <0.05 was considered statistically significant.

## Results

### Baseline Characteristics of RA Patients and OA Patients

As illustrated in Table 1, RA patients were stratified into active patients (DAS28, mean±SD, 4.94±0.95) and inactive patients (DAS28, 2.90±0.30). As expected, active RA patients had higher levels of serum CRP and daily doses of oral corticosteroids compared to inactive RA patients. There were no significant differences between active RA patients and inactive RA patients regarding baseline demographic data, positive rates of RF and anti-CCP antibodies, or the proportions of DMARDs used. There were no significant differences in demographic data between RA patients and OA patients.

**Table 1:** Clinical characteristics and laboratory findings in patients with active rheumatoid arthritis (RA), inactive RA, patients with knee osteoarthritis (OA) and healthy controls (HC).

	Active RA (n = 22)	Inactive RA (n = 16)	Knee OA (n = 12)	HC (n = 10)
Mean age (years)	50.3±10.4	56.9±17.5	58.3±5.6	52.0±8.3
Female (%)	18 (81.8%)	12 (75.0%)	10 (83.3%)	7 (70.0%)
RF positivity (%)	16 (72.7%)	12 (75.0%)	NA	NA
Anti-CCP positivity (%)	15 (68.2%)	10 (62.5%)	NA	NA
CRP (mg/dl)	2.54±1.91*	0.43±0.25	NA	NA
DAS-28	4.94±0.95*	2.90±0.30	NA	NA
Daily steroid dose (mg)	5.2±2.0*	2.5±1.3	NA	NA
DMARDs used				
Methotrexate	19 (86.4%)	11 (68.8%)	NA	NA
Sulfasalazine	17 (77.3%)	10 (62.5%)	NA	NA
Hydroxychloroquine	16 (72.7%)	8 (50.0%)	NA	NA
Cyclosporine	4 (18.2%)	0 (0.0%)	NA	NA

Data are presented as mean ± SD or number (percentage); NA: not applicable.

RF: rheumatoid factor; Anti-CCP: anti-cyclic citrullinated peptide antibodies; CRP: C-reactive protein; DAS28: disease activity score for 28-joints; DMARDs: disease-modifying anti-rheumatic drugs.

A result was considered positive for RF when the concentration was ≧15 IU/ml; a result was considered positive for anti-CCP antibodies if the titer was≧20 IU/ml. *p<0.001, versus inactive RA group, determined using the Mann-Whitney U test.

### Percentages of Circulating MDL-1-expressing Monocytes

**Figure 1:**
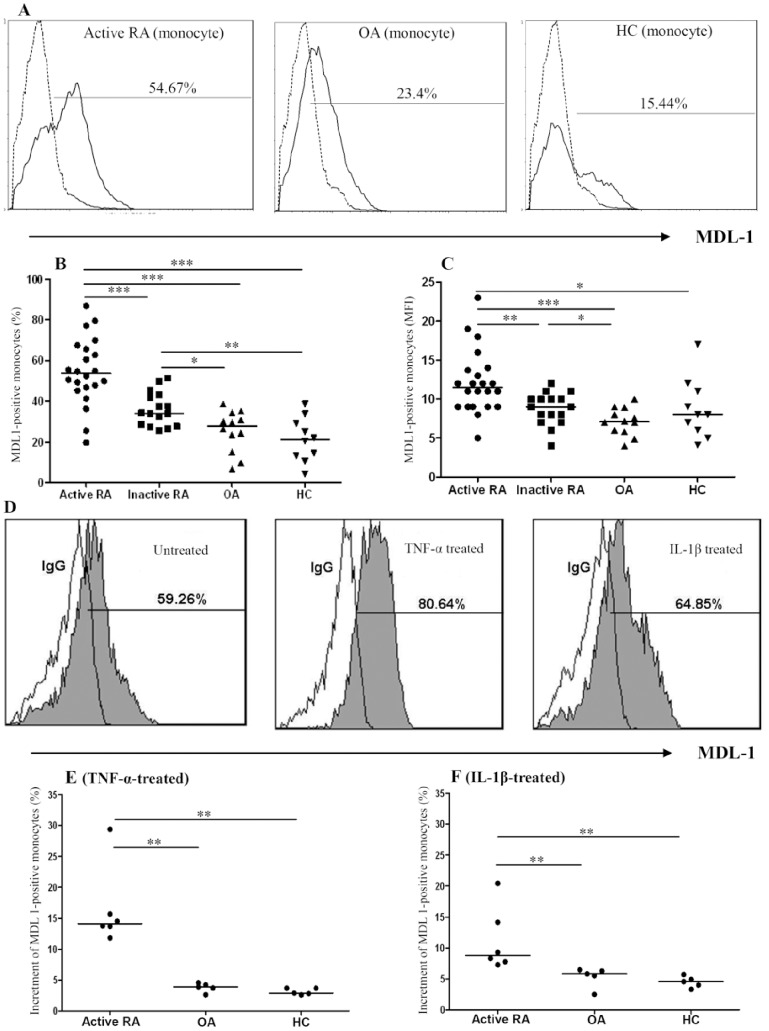
Representative examples of flow cytometric histograms (A) of MDL-1-expressing monocytes obtained from peripheral blood of one active RA patient, one OA patient, and one healthy subject. Comparisons of the percentages and mean fluorescence intensity (MFI) of circulating MDL-1-expressing monocytes are shown for 22 active RA patients, 16 inactive RA patients, 12 OA patients, and 10 healthy control subjects (B and C respectively). To evaluate the effects of TNF-α or IL-1β on MDL-1 expression, we examined changes in the percentages of circulating MDL-1-expressing monocytes in active RA patients, OA patients and healthy subjects. Representative examples of flow cytometric histograms (D) of MDL-1-expressing monocytes obtained from one active RA patient after stimulation with TNF-α or IL-1β. Comparisons of the increment of the percentages of MDL-1-expressing monocytes after stimulation with TNF-α and IL-1β are shown for 6 active RA patients, 5 OA patients, and 5 healthy subjects (E and F respectively). The middle horizontal line indicates median value for each group. *p<0.05, **p<0.01, ***p<0.001, as determined by Mann-Whitney U test.

Representative examples of flow cytometric histograms of MDL-1 expression on monocytes obtained from one active RA patient, one OA patient, and one healthy volunteer are shown in Figure 1A. Significantly higher percentages of MDL-1-expressing monocytes were observed in active RA patients compared to inactive RA patients, OA patients, or healthy control subjects (Figure 1B and Table 2). Similarly, significantly higher mean fluorescence intensity (MFI) of MDL-1 staining on monocytes was observed in active RA patients compared to inactive RA patients, OA patients, or healthy control subjects (Figure 1C). The percentages of MDL-1-expressing monocytes were significantly higher in inactive RA patients than in OA patients, or healthy control subjects. However, there was no significant difference in the percentages or MFI of MDL-1-expressing monocytes between early RA patients (n = 8) and late RA patients (n = 30), or between OA patients and healthy control subjects.

**Table 2:** Levels of circulating MDL-1-expressing monocytes, mRNA expression levels of MDL-1 and DAP12 on PBMCs, and levels of serum proinflammatory cytokines in patients with active rheumatoid arthritis (RA), patients with inactive RA, patients with osteoarthritis (OA) and healthy controls (HC).

MDL-1 and cytokines, patient groups	Median (interquartile range)
MDL-1-expressing monocytes (%)	
Active RA	53.6 (46.4–66.1)***^###§§§^
Inactive RA	34.1 (27.9–42.7)**^#^
OA	27.9 (17.1–33.5)
HC	21.2 (12.6–30.2)
MDL-1-expressing monocytes (MFI)	
Active RA	11.5 (9.0–13.8)*^###§§^
Inactive RA	9.0 (7.3–10.0)^#^
OA	7.1 (5.8–8.7)
HC	8.0 (5.8–11.3)
MDL-1 transcript levels on PBMCs	
Active RA	1.64 (1.31–2.38)***^###§§§^
Inactive RA	0.89 (0.64–1.36)^#^
OA	0.71 (0.50–1.24)
HC	0.99 (0.44–1.29)
DAP12 transcript levels on PBMCs	
Active RA	1.53 (1.09–1.74)**^##§§^
Inactive RA	1.06 (0.92–1.21)^#^
OA	0.77 (0.54–1.09)
HC	0.90 (0.67–1.26)
Interleukin-1β (pg/ml)	
Active RA	9.28 (7.88–18.49)***^##^
Inactive RA	8.93 (8.17–12.84)***^ ##^
OA	7.53 (6.09–7.94)
HC	4.39 (2.54–7.15)
Interleukin-6 (pg/ml)	
Active RA	1530.1 (921.3–2004.0)***^ §§^
Inactive RA	754.0 (543.7–1333.2)***
OA	920.9 (476.0–1798.2)***
HC	74.7 (52.7–94.4)
Interleukin-17A (pg/ml)	
Active RA	145.79 (101.83–273.34)*
Inactive RA	114.38 (92.46–181.14)
OA	131.70 (86.81–202.85)
HC	86.57 (51.74–181.08)
Tumor necrosis factor-α (pg/ml)	
Active RA	142.37 (80.45–322.80)***^ ###^
Inactive RA	95.28 (34.40–155.10)**^ #^
OA	39.54 (16.00–66.46)
HC	24.30 (17.70–35.38)

MDL-1: Myeloid DAP12-associated lectin-1; MFI: mean fluorescence intensity; DAP12: DNAX adaptor protein 12; PBMCs: peripheral blood mononuclear cells; *p<0.05, **p<0.01, ***p<0.001, vs. HC; ^#^p<0.05,^ ##^p<0.01, ^###^p<0.001, vs. OA;

§ p<0.05,^ §§^p<0.01, ^§§§^p<0.001, vs. inactive RA group, were determined by Mann-Whitney U test.

In addition, we examined whether TNF-α or IL-1β could affect MDL-1 expression; our results showed that TNF-α stimulation of PBMCs from RA patients induced a higher increment of MDL-1-expressing monocytes (median 14.1%, interquartile range [IQR] 13.2–19.1%) compared with stimulation of PBMCs from OA patients (median 3.9%, IQR3.2–4.4%, p<0.01) or healthy controls (median 2.9%, IQR2.7–3.7%, p<0.01; Figure 1E). Similarly, IL-1β stimulation of PBMCs from RA patients induced a higher increment of MDL-1-expressing monocytes (median 8.8%, IQR7.6–15.7%) compared with stimulation of PBMCs from OA patients (median 5.8%, IQR4.0–6.4%, p<0.01) or from healthy control subjects (median 4.6%, IQR3.7–5.3%, p<0.01; Figure 1F).

### mRNA Expression Levels of MDL-1and DAP12 Using qPCR

**Figure 2:**
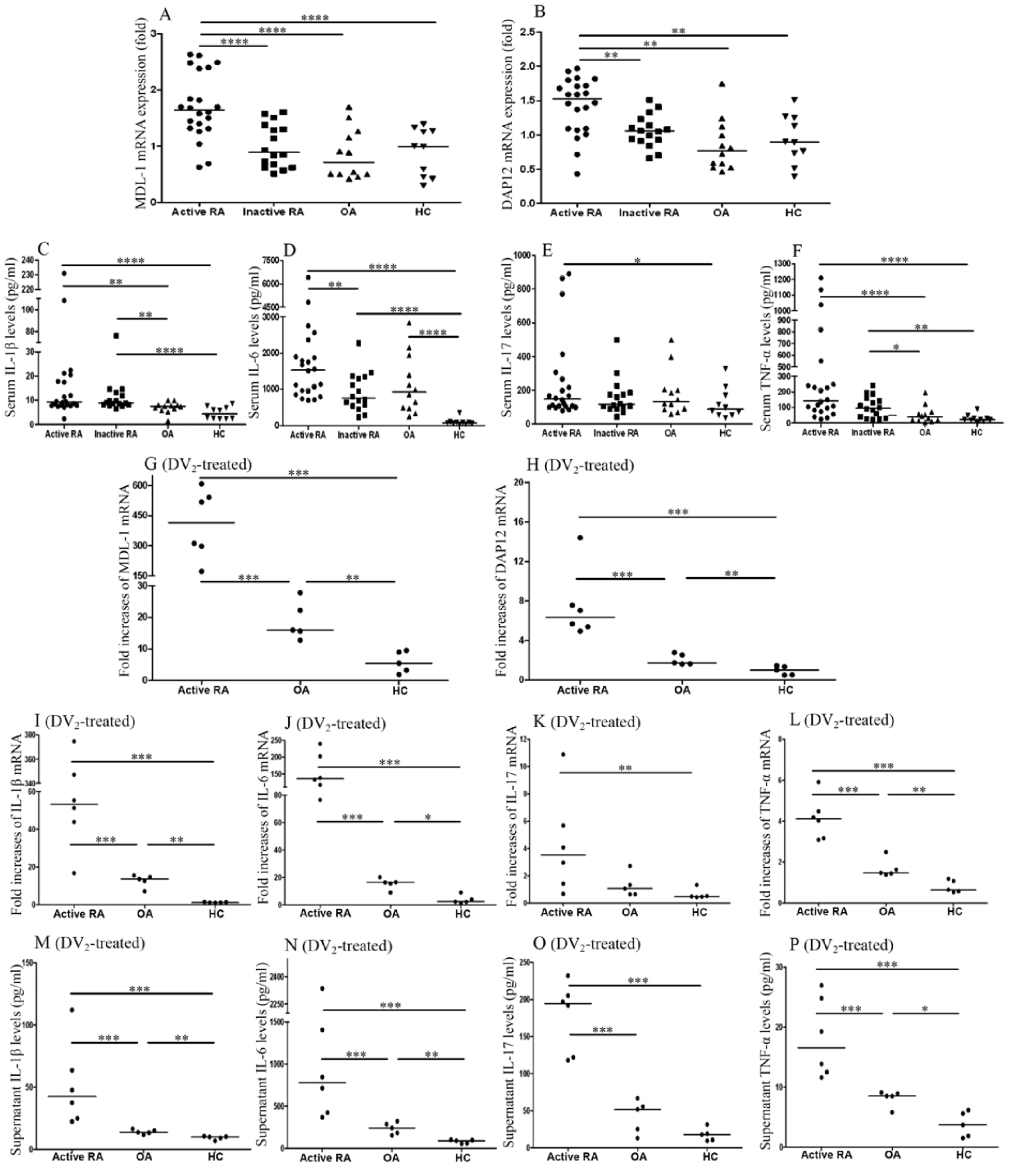
Comparisons of relative expression levels of MDL-1 and DAP12 mRNA on peripheral blood mononuclear cells (A, B; respectively) and serum levels of IL-1β, IL-6, IL-17A, and TNF-α, (C, D, E, F; respectively) are shown for 22 active RA patients, 16 inactive RA patients, 12 OA patients, and 10 healthy subjects. To explore the functional role of MDL-1 activation, we examined the fold-increases of mRNA expression levels of MDL-1 and DAP12 (G, H; respectively), and the fold-increases of mRNA expression levels and supernatant levels of downstream cytokines, including IL-1β, IL-6, IL-17A, and TNF-α (I–P) on PBMCs treated with MDL-1 activator (Dengue virus type 2 [DV2]-antigen) in 6 active RA patients, 5 OA patients, and 5 healthy controls. The horizontal line indicates median value for each group. *p<0.05, **p<0.01, ***p<0.005, ****p<0.001, as determined by Mann-Whitney U test.

As shown in Figures 2A–2B and Table 2, significantly higher levels of MDL-1 and DAP12 mRNA expression were found on PBMCs from active RA patients compared to PBMCs from inactive RA patients, OA patients, or healthy controls. However, there was no significant difference in mRNA expression levels of MDL-1 or DAP12 between early RA patients and late RA patients, between inactive RA patients and OA patients, or between OA patients and healthy control subjects.

### Serum Levels of Proinflammatory Cytokines

As shown in Figures 2C–2F and Table 2, levels of serum IL-1β, IL-6, IL-17A, and TNF-α were significantly higher in active RA patients compared to healthy subjects. Active RA patients had significantly higher IL-6 levels than those in inactive RA patients, and also had higher levels of IL-1β and TNF-α compared to OA patients. Inactive RA patients had significantly higher levels of IL-1β, IL-6, and TNF-α compared to healthy controls, and higher levels of IL-1β and TNF-α compared to OA patients. OA patients had significantly higher levels of IL-6 compared to healthy controls. However, there was no significant difference in serum levels of IL-1β, IL-17A, and TNF-α between OA patients and healthy control subjects.

### Functionality of MDL-1-mediated Production of Proinflammatory Cytokines

We examined whether the enhanced MDL-1expression was functional in terms of DAP12 expression and proinflammatory cytokine production. Our results showed that DV2-antigen stimulation of PBMCs from active RA patients induced greater folds increases of MDL-1 (median, 414.8, IQR 267.0–557.6), DAP12 (median, 6.4, IQR 5.3–9.3), IL-1β (median, 53.3, IQR 37.0–354.1), IL-6 (median, 135.4, IQR 106.7–211.5), IL-17A (median, 3.5, IQR 1.2–7.0), and TNF-α (median, 4.1, IQR 3.1–4.8) compared to stimulation of PBMCs from OA patients (median, 16.0, IQR 14.2–25.0, p<0.005; 1.7, IQR 1.6–2.7, p<0.005; 13.6, IQR 9.8–15.1, p<0.005; 16.4, IQR 12.3–18.3, p<0.005; 1.1, IQR 0.6–2.0, p = 0.052; and 1.5, IQR 1.4–2.1, p<0.005; respectively) or from healthy control subjects (median, 5.5, IQR 2.6–9.3, p<0.005; 1.0, IQR 0.5–1.4, p<0.005; 1.1, IQR 1.0–1.4, p<0.005; 2.3, IQR 1.7–6.5, p<0.005; 0.5, IQR 0.5–0.9, p<0.01; and 0.6, IQR 0.6–1.1, p<0.005; respectively, Figures 2G–2L). Similarly, we demonstrated that DV2-antigen stimulation of PBMCs from active RA patients induced higher levels of supernatant IL-1β (median, 42.5 pg/ml, IQR 24.2–75.8 pg/ml), IL-6 (median, 779.6 pg/ml, IQR 408.9–1637.9 pg/ml), IL-17A (median, 194.5 pg/ml, IQR 121.0–211.9 pg/ml), and TNF-α (median, 16.6 pg/ml, IQR 12.3–25.4 pg/ml) compared to stimulation of PBMCs from OA patients (median, 13.6 pg/ml, IQR 12.6–15.7 pg/ml, p<0.005; 241.5 pg/ml, IQR 168.2–303.9 pg/ml, p<0.005; 51.8 pg/ml, IQR 19.1–61.1 pg/ml, p<0.005; and 8.5 pg/ml, IQR 7.1–9.0 pg/ml, p<0.005; respectively) or from healthy control subjects (median, 9.9 pg/ml, IQR 7.9–10.3 pg/ml, p<0.005; 88.5 pg/ml, IQR 58.7–98.9 pg/ml, p<0.005; 17.8 pg/ml, IQR 9.9–25.3 pg/ml, p<0.005; and 3.7 pg/ml, IQR 1.7–5.9 pg/ml, p<0.005; respectively, Figures 2M–2P).

### Expression Levels of MDL-1 and DAP12 on Synovial Membranes Using IHC

**Figure 3:**
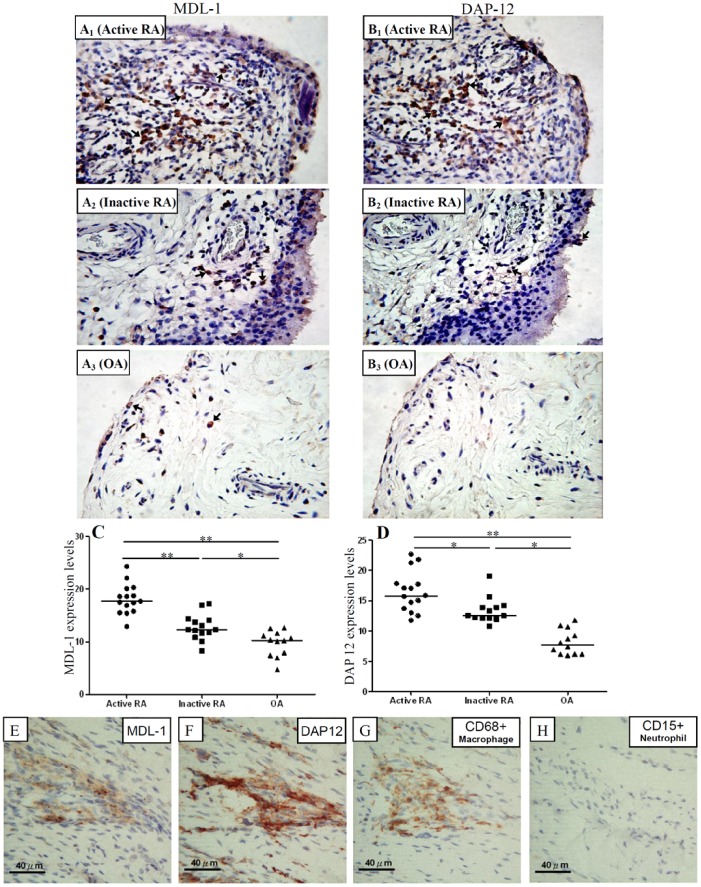
Representative examples of immunostaining with MDL-1 and DAP12 (arrows, original magnification x 200) obtained from synovial membranes of one active RA patient (A1 and B1; respectively), one inactive RA patient (A2 and B2; respectively), one OA patient (A3 and B3; respectively). Comparisons of expression levels of MDL-1 and DAP12 on synovial membranes (C and D; respectively) are shown for 15 active RA patients, 14 inactive RA patients, and 12 OA patients. The horizontal line indicates median value for each group. *p<0.005, **p<0.001, as determined by Mann-Whitney U test. We also demonstrated that strongly positive staining of MDL-1 and DAP12 were expressed on CD68+ macrophages, not on CD15+ neutrophils in serial sections of synovial biopsy specimens from one RA patient (Figures 3E–3H).

Representative examples of immunostaining for MDL-1 and DAP12 on synovial biopsy specimens from one active RA patient, one inactive RA patient, and one OA patient are shown in Figure 3. There were higher expression levels of MDL-1 and DAS12 protein in synovial membranes from active RA patients (median 17.70, IQR 15.80–20.10 and 15.80, IQR 13.70–17.80; respectively) compared to levels in SMs from inactive RA patients (12.30, IQR 11.40–14.18, p<0.05 and 12.55, IQR 12.10–13.98, p<0.05; respectively) or from OA patients (10.25, IQR 7.53–11.48, p<0.005 and 7.75, IQR 6.20–10.33, p<0.001; respectively). We also observed significantly higher expression levels of MDL-1 and DAS12 protein in SMs from inactive RA patients compared to those from OA patients (p<0.05 and p<0.005; respectively). In addition, we found that strongly positive staining of MDL-1 and DAP12 were expressed on CD68+ macrophages, not on CD15+ neutrophils in serial sections of SMs (Figures 3E–3H). However, there are limitations of protein staining assessment when using this approach, such as accounting for nonspecific staining and identifying cell associated staining and vascular staining.

### Correlations of MDL-1 Expression Levels with Disease Activity Parameters, Erosion Scores, Joint Space Narrowing Scores, and DAP12 Expression Levels in RA Patients

As illustrated in Table 3, MDL-1 expression levels were positively correlated with DAS28, CRP levels, erosion scores, and JSN scores in RA patients. The percentages of circulating MDL-1-expressing monocytes and mRNA expression levels of MDL-1 were positively correlated with serum levels of IL-1β, IL-6, IL-17A, and TNF-α in RA patient. Moreover, MDL-1 expression levels were positively correlated with DAP12 expression levels in synovial membranes from RA patients.

**Table 3:** Correlation between expression levels of MDL-1 and disease activity parameters as well as cytokine levels in 38 patients with rheumatoid arthritis.

Parameters	MDL-1 expression on monocytes	MDL-1 mRNA levels on PBMCs	MDL-1 protein levels on SM
DAS28	0.392*	0.428**	0.613***
CRP, mg/dl	0.289*	0.395*	0.596**
Erosion score	0.569**	0.641***	0.546**
JSN score	0.476**	0.484**	0.393*
IL-1β, pg/ml	0.437**	0.274*	0.389*
IL-6, pg/ml	0.468**	0.387*	0.410*
IL-17A, pg/ml	0.262*	0.280*	0.135
TNF-α, pg/ml	0.605***	0.459**	0.321
DAP12 mRNA levels on PBMCs	0.656***	0.702***	0.447*
DAP12 protein levels on SM	0.119	0.207	0.462**

MDL-1: Myeloid DAP12-associated lectin-1; DAP12: DNAX adaptor protein 12; PBMCs: peripheral blood mononuclear cells; SM: synovial membranes; DAS28: disease activity score for 28-joints; CRP: C-reactive protein; JSN score: joint space narrowing score; IL-1β: interleukin-1β; IL-6: interleukin-6; IL-17A: interleukin-17A; TNF-α: tumor necrosis factor-α.

* p<0.05, **p<0.005, ***p<0.001 were obtained by the nonparametric Spearman’s correlation test.

### Changes in Percentages of Circulating MDL-1-expressing Monocytes and mRNA Expression Levels of MDL-1 and DAP12 on PBMCs from RA Patients after Therapy

**Figure 4:**
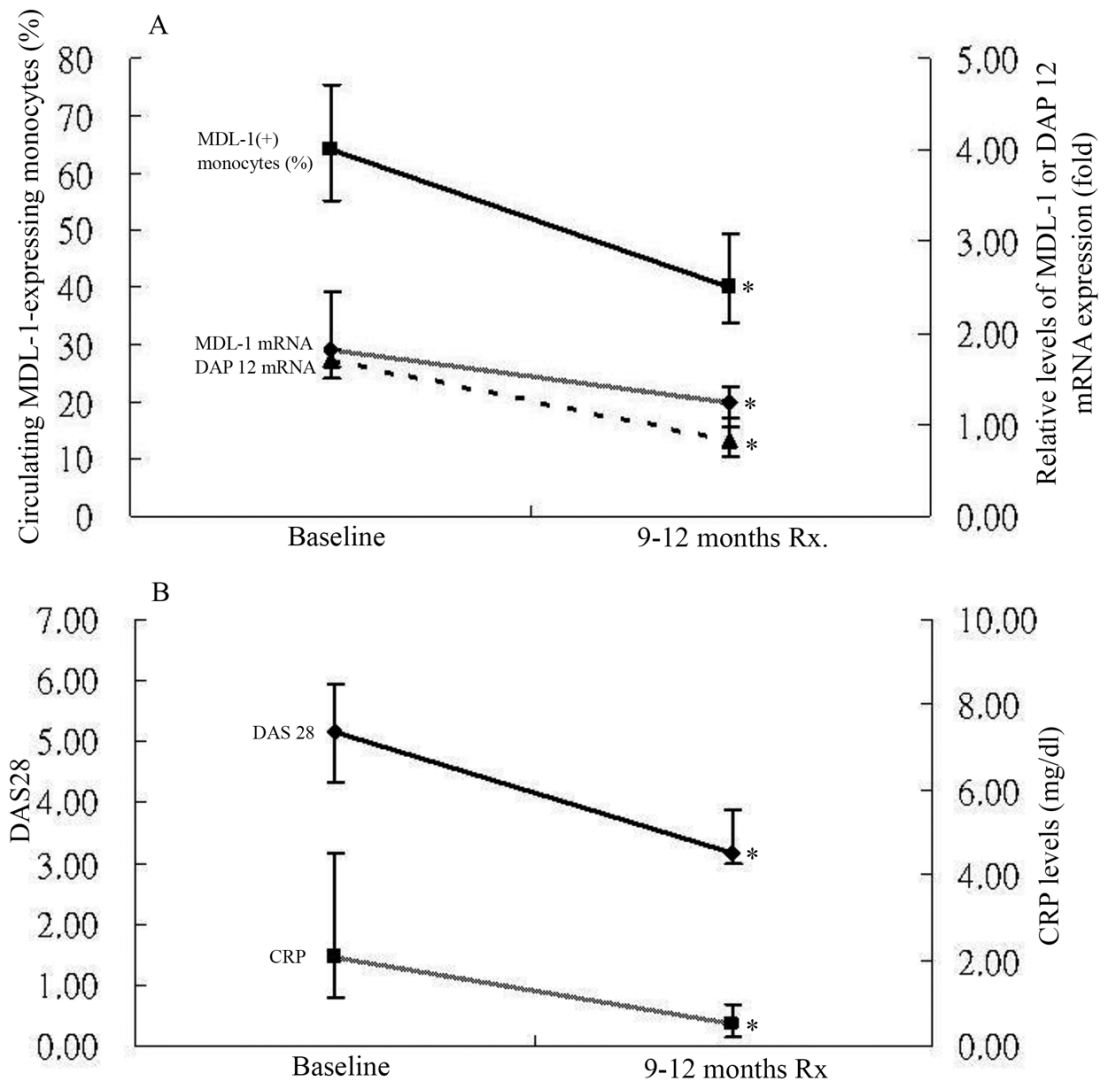
Changes in (A) percentages of circulating MDL-1-expressing monocytes and mRNA expresison levels of MDL-1 and DAP12, and changes in (B) disease activity score for 28-joints (DAS28) and C-reactive protein (CRP) levels in 12 RA patients at active phase (at baseline) and after 9–12 months of therapy. Data are presented as mean ± SEM. *p<0.005, versus active phase, determined by the Wilcoxon signed rank test.

Twelve active RA patients were available for examination of their expression levels of MDL-1 and DAP12 in the active phase (baseline time-point) and after 9–12 months of therapy with triple DMARDs, including methotrexate, hydroxychloroquine, and sulfasalazine. As shown in Figure 4, the percentages of MDL-1-expressing monocytes significantly decreased (mean ± standard error of the mean; 65.22±3.35% vs. 40.83±2.38%, p<0.005), paralleling the decreases in DAS28 (5.15±0.27 vs. 3.35±0.24, p<0.005) and CRP (2.93±0.64 mg/dl vs. 0.70±0.19 mg/dl, p<0.005). Similarly, the relative expression levels of MDL-1 and DAP12 mRNA also significantly declined (1.98±0.13 vs. 1.20±0.08; 1.65±0.07 vs. 0.85±0.06, both p<0.005), paralleling the decreases in DAS28 and CRP.

## Discussion

MDL-1, a novel member of the C-type lectin family, has been shown to be involved in murine joint inflammation [22]. However, the role of MDL-1 in the pathogenesis of human RA remains unclear. The present study is the first attempt to characterize the expression of MDL-1 and its associated adaptor-DAP12 in RA patients. Our results show that MDL-1 is highly expressed on circulating monocytes in active RA patients, consistent with the findings of an increased expression of MDL-1 on murine bone marrow macrophages [22], and the involvement of MDL-1 in the activation of myeloid cells [20] in other recent studies. In addition, our data demonstrated significantly higher percentages and MFI of circulating MDL-1-expressing monocytes in active RA patients compared to inactive RA patients and OA patients. The results are similar to those of another study showing enhanced percentages of MDL-1-expressing monocytes following arthritis induction [22]. We also showed that the percentages of circulating MDL-1-expressing monocytes were positively correlated with DAS28, erosion scores, and JSN scores in RA patients, supporting the previous findings of that MDL-1 is a key regulator of synovitis and bone erosion during progression of autoimmune arthritis [22]. Moreover, the percentages of MDL-1-expressing monocytes significantly decreased after effective therapy in our RA patients. These observations strongly suggest that MDL-1 overexpression plays a major role in the pathogenesis of human RA.

Since Joyce-Shaikh observed that TNF-α could promote MDL-1 expression in murine arthritis [22], we examined the role of TNF-α or IL-1β in the regulation of MDL-1 expression. The *ex vivo* study demonstrated that TNF-α or IL-1β stimulation on PBMCs from our active RA patients resulted in significantly enhanced levels of MDL-1 expression when compared to those from OA patients or from healthy control subjects. Therefore, there still exists the possibility that MDL-1 upregulation may represent an epiphenomenon of rheumatoid inflammation rather than serve as a primary event in the pathogenesis of human RA.

To verify MDL-1 expression at the mRNA level in human RA, qPCR for MDL-1 gene expression on PBMCs was performed for our RA patients and control groups. We demonstrated that relative gene expression levels of MDL-1 were significantly higher in active RA patients when compared to inactive RA patients, OA patients, or healthy controls. Moreover, the gene expression levels of MDL-1 were positively correlated with DAS28, CRP levels, erosion scores, and JSN scores in RA patients. Such results provide robust evidence of MDL-1 overexpression in active RA patients. However, studies with large cohorts of RA patients should be conducted to confirm our findings.

To confirm the production of MDL-1 in inflamed joint tissues, we investigated MDL-1 expression levels in SMs from RA patients and OA patients by IHC. The results showed significantly higher levels of MDL-1 protein in SMs from active RA patients in comparison to levels in SMs from inactive RA patients or OA patients; this was consistent with previous findings of that MDL-1 was expressed on inflamed joints in collagen-induced arthritis [22]. Besides, as revealed in another study that MDL-1 expression co-localizes with a subset of CD68+ macrophages [16], we found strongly positive staining of MDL-1 and DAP12 expressed on CD68+ macrophages, not on CD15+ neutrophils (Figure 3E–3H). Moreover, we demonstrated that MDL-1 expression levels in SMs from RA patients were positively correlated with DAS28, erosion scores, and JSN scores. Our data support the previous findings of that mice treated with MDL-1 agonist had higher arthritis scores than the isotype controls, whereas MDL-1 blockade markedly reduced arthritis and bone erosion [22]. These observations imply that MDL-1 overexpression might be a novel biomarker for disease activity and a potential therapeutic target.

MDL-1 interacts with the ITAM-bearing adaptor protein DAP12 [21], which activate the Syk-signaling pathway, leading to synovial inflammation and bone erosion [4]. Our results provide the first evidence that both MDL-1 and DAP12 were highly expressed on PBMCs at the gene expression level, and also in SMs at the protein level in active RA patients, indicating the potential involvement of MDL-1/DAP12 in RA pathogenesis. Similarly, DAP12 expression levels in synovial tissue have been found to be significantly higher in active RA patients than those in inactive RA patients or OA patients [19]. Moreover, we demonstrated a positive correlation not only between mRNA expression levels of MDL-1 and those of DAP12 on PBMCs, but also between the protein expression levels of MDL-1 and those of DAP12 in the SMs from RA patients. Such results support the findings that MDL-1 expression is coupled with expression of DAP12, which acts as a signaling molecule for the MDL-1 receptor [21]. In the *ex vivo* study, we also demonstrated a six-fold increase of DAP12 mRNA expression levels on PBMCs treated with MDL-1 activator. These observations support the recent finding that DAP12 gene expression levels on myeloid cells were elevated in MDL-1 agonist-treated mice, whereas MDL-1 blockade suppressed DAP12 expression [22]. In addition, MDL-1 has been shown to associate with DAP12 to form receptor complexes involved in monocytic activation and inflammatory responses [36]–[37]. These findings suggest potential links between MDL-1 and DAP12, and support the observations of a critical role of MDL-1/DAP12 complex in the pathogenesis of arthritis and osteoclastogenesis [22], [38]. Involvement of the MDL-1/DAP12 signaling pathway in RA pathogenesis was reinforced by the finding that the severity of arthritis was markedly lower in both DAP12^–/–^ and MDL-1^–/–^ mice when compared to wild-type mice [22]. However, in our study, MDL-1 mRNA expression levels on PBMCs were not in parallel with DAP12 protein expression levels in the SMs from RA patients. Probable reasons for this result include the small sample size and that MDL-1/DAP12 coupling signaling occurs mainly in inflamed tissues.

In agreement with the recent finding that MDL-1 cross-linking could trigger the secretion of proinflammatory cytokines [22], we found that the elevated levels of serum IL-1β, IL-6, IL-17A, and TNF-α were positively correlated with MDL-1 expression levels in RA patients. Moreover, we investigated the functional relation between MDL-1 activation and the downstream proinflammatory cytokines. The results show that MDL-1 activator (DV2-antigen) stimulation on PBMCs induced greater-fold increases of mRNA expression levels of IL-1β (up to ∼53-fold), IL-6 (up to ∼135-fold), IL-17A (up to ∼3.5-fold), and TNF-α (up to ∼4-fold) in active RA patients compared to those in OA patients or healthy subjects, indicating that MDL-1 activation is functional. Such results support another study showing that treatment with MDL-1 agonist enhanced the expression of IL-1β, IL-6, IL-17A and TNF-α; whereas MDL-1 blockade suppressed cytokine gene expression [22]. Up-regulated expression of MDL-1 together with elevated levels of proinflammatory cytokines may have pathogenic relevance by representing a potential mechanism of amplification of synovial inflammation in active RA patients.

There are some limitations of our study. This was a preliminary study that enrolled a limited number of active RA patients. In addition, we did not investigate the role of DAP10 in the pathogenesis of human RA because of the different associated receptors, signaling kinase, and intracellular domain [36], [39]. Because most of patients enrolled in our study were not in early RA stage, our results might not be directly applicable to early RA patients. Therefore, a long-term study enrolling a larger group of patients, including an early RA population, is required for validation of our findings.

## Conclusion

Our results show that MDL-1 and its associated adaptor-DAP12 were concordantly overexpressed on PBMCs and the inflamed synovium in active RA patients. We also provide the first evidence that MDL-1 upregulation may have a possible link with DAP12 and inflammatory responses in human RA. The preliminary results might partly explain the role of MDL-1-linked signaling in the inflammatory cascade of RA. Such studies may be of translational interest because they may provide further evidence that can be used to develop potentially therapeutic modalities for RA, including the use of MDL-1 and Syk kinase inhibitors [22], [40]–[41].
